# How Much Superior Rectus Underaction is Considered Normal?

**DOI:** 10.22599/bioj.159

**Published:** 2021-01-21

**Authors:** Bethany Shaw, Charlotte Codina, Sonia Toor

**Affiliations:** 1 Northern Lincolnshire and Goole NHS Foundation Trust, GB; 2 Division of Ophthalmology and Orthoptics, University of Sheffield, GB

**Keywords:** Ocular movements, Superior rectus, Synoptophore

## Abstract

**Purpose:**

It is considered normal to have a small amount of superior rectus weakness in laevo and dextro elevation; however, there is no documented definition for these normal parameters within a healthy young adult population using ocular movement testing and the synoptophore. The aim of this study was to collect normative data on the degree of superior rectus underaction in healthy young adults.

**Method:**

Twenty-nine healthy adults (3 males and 26 females, mean age 20.30 ± 1.70 years) were recruited. Superior recti underactions and inferior oblique overactions were recorded during routine ocular movement testing and mean and median values calculated. Subjective horizontal, vertical and torsional measurements were taken in degrees on the synoptophore in primary position, laevo elevation and dextro elevation.

**Results:**

Most participants (79.31%) had some degree of observable superior rectus underaction in either eye or in both eyes on ocular movement testing (mean superior rectus underaction of –0.69 units in laevo elevation and –0.71 units in dextro elevation, range = –1.5 to –0.5 units; median –1 units, interquartile range (IQR) = –1 to –1 units). Most participants (62.07%) had some degree of superior rectus underaction in either eye or in both eyes on the synoptophore (mean left and right superior recti underactions of –0.48 degrees, range = –3 to –1 degrees; median 0 degrees, IQR = –1 to –1 degrees).

**Conclusion:**

The majority of young healthy adults in this study showed some degree of superior rectus underaction. On ocular movement testing, –0.70 units of underaction, and on the synoptophore, –0.48 degrees of underaction are the mean levels of weakness to be expected. Superior rectus underactions greater than –1 units for ocular movement testing and –1 degrees on the synoptophore in healthy young adults should be carefully evaluated, together with other important clinical signs.

## INTRODUCTION

Anecdotally, orthoptists note that many adults with otherwise full ocular movements display mild superior rectus underactions on ocular movement testing, typically identified in laevo and dextro elevation. It is important to identify the normal parameters of superior rectus underaction in order to ensure accuracy in detecting pathology, such as a superior rectus palsy, as well as to prevent unnecessary further investigations.

Superior rectus muscle function is routinely assessed during ocular movement testing, where the patient fixates a light in primary position then follows the light into eight positions of gaze. The relative corneal reflection positions are compared with those revealed in extreme gaze. The superior rectus is best examined in elevation in abduction where an underaction of the abducting eye can be observed against an overaction of the elevating adducting eye on alternate cover testing (Ansons & Davis 2014).

When differentially diagnosing a palsy of the superior rectus against an underaction considered to be within normal limits, clinicians may rely on observing additional levator palpabrae superioris (LPS) underactions that are expected with this condition: the close proximity of the LPS and superior rectus axons mean that an acquired pathology usually involves both muscles (Bienfang 1975). However, Mims (2011) discussed findings from three children who had isolated superior rectus palsies without a ptosis, which were believed to be congenital in origin, possibly due to hypoplastic superior recti muscles. Therefore, associated LPS dysfunction cannot always be relied on to differentiate between a non-significant superior rectus underaction and a significant neurological palsy. Clark and Isenberg (2001) and Davidson and Knox (2002) both suggested that asymmetry of the superior recti function is the most accurate way of identifying pathology.

Ocular elevation is known to be reduced in the elderly (Chamberlain 1970; Clark & Isenberg 2001; Davidson & Knox 2002). Davidson and Knox (2002) assessed the range of ocular movements (binocularly and uniocularly) in 10 young participants (mean age 20.2 ± 1.7 years) and 12 older participants (mean age 72.72 ± 6.1 years). The extent of binocular elevation was significantly less in the older group (mean excursion of 26.19 ± 5.4 degrees) than in the younger group (29.83 ± 0.5 degrees).

Similarly, Chamberlain (1970) found a gradual increase in the restriction of elevation with increasing age. Participants, aged 5 to 94 years, were assessed monocularly using the arc of Schweiger hand perimeter. They found that the normal upward rotation was 40 degrees for 5–14 year olds, 33 degrees for 35–44 year olds and only 16 degrees for both 75–84 year olds and 85–94 year olds—a 60% decrease from the youngest age group. Chamberlain suggested the decrease in elevation with age resulted from a decreased necessity to look up with increasing age, causing the muscles to become weaker from reduced activity.

Haggerty et al (2005) carried out uniocular field of fixation examinations in 35 healthy adults (20 to 60 years) to find the normal excursions of each extraocular muscle using the Goldmann Perimeter. Contrary to the studies previously mentioned, Haggerty noted no significant age related decline for the superior rectus excursion. The 20–29 years age group had a mean upward excursion of 44.6 degrees and the 40–49 years age group had a smaller mean excursion of 40.6 degrees. However, the 50–59 years age group had a larger mean upward excursion of 42.9 degrees, disputing the suggestion that upward ocular excursion decreases with increasing age.

Clark and Isenberg (2001) measured the underactions on binocular versions rather than monocular limitations. They reviewed 124 participants, aged 23 to 84 years, undergoing a standardised lateral version light-reflex test (Urist 1967) to quantify normal maximum versions. They reported that from the third to the ninth decade the maximum versions into extremes of gaze decrease by 0.50% to 1% of ocular rotation each consecutive year, with the most affected position of gaze being elevation and the least affected being depression. They did not measure superior recti underactions or overactions in laevo and dextro elevation and so did not provide a true representation for the function of the superior rectus.

Although data exists as to the maximum excursions of the superior recti in various age groups, this has never before been compared with the orthoptic assessment of ocular movements in nine positions of gaze. The superior recti are evaluated in laevo and dextro elevation as part of this assessment, and orthoptists frequently note slight superior recti underactions and estimate whether or not this is within normal limits. This study collected normative data using ocular movement testing and the synoptophore to determine the typical ocular movement limits of rotation in laevo and dextro elevation in healthy young adults with no known ocular movement disorders.

## METHOD

The study adhered to the Declaration of Helsinki. Ethical approval was obtained from the University of Sheffield Ethics Committee and written informed consent was obtained from all participants.

Orthoptic students aged 18 to 24 years old were recruited from The University of Sheffield. All testing was conducted by one examiner (BS) to ensure consistency in measurements. Participants had no known ocular pathology, no manifest strabismus and no ocular movement defects or history of these. The corrected visual acuity was assessed monocularly using a logMAR chart at 3m with use of the termination rule. Vision criteria for entry to the study was corrected visual acuity with glasses or contact lenses of at least 0.20 logMAR and Frisby stereopsis of 150 seconds of arc.

The ocular movements in free space were always assessed prior to synoptophore measurements and documented for each participant in nine positions of gaze, in line with standard clinical practice as described by Vivian and Morris (1993). The superior rectus was assessed in elevation at 23 degrees of abduction away from the medial plane as per Ansons and Davis (2014). Accurate angle estimation was achieved by prior practice. Under and over actions of the extraocular muscles were recorded to the nearest +/–0.5 unit on a scale of 0 to 4 units (0 indicating no under or over action and 4 indicating complete under or over action). Ocular movements in laevo and dextro elevation were carefully documented and used for the analysis. Other positions were assessed for the purpose of excluding pathology.

Participants were assessed for any latent deviation in primary position prior to other positions of gaze on the synoptophore, which is an orthoptic instrument for assessing ocular deviations and binocularity in different positions of gaze. Measurement of deviations in laevo and dextro elevation (upwards to the left and upwards to the right) were made relative to those found in primary position using the foveal Maddox slides. The fixing eye (the eye behind the tube in the locked position) viewed the circle, and the non-fixing eye (the eye behind the tube being moved) viewed the cross. The eyes were positioned at 20 degrees of elevation and 23 degrees of abduction. Subjective vertical, horizontal and torsional measurements were taken to the nearest +/–0.5 degrees. Subjective measurements of deviation were chosen for maximum accuracy, as measurements were very small in this normative population and small torsional deviations could only be detected and measured this way. Counterbalancing by Latin square was used in order to determine the sequence in which measurements on the synoptophore were taken for each participant so that the same position of gaze was not assessed last each time. This minimised any order effects by preventing each participant from potentially fatiguing in the same position of gaze.

## RESULTS

Thirty-one participants were recruited and two participants were excluded: one due to previous treatment for convergence insufficiency and another due to the presence of a mild ocular movement defect. The remaining 29 participants (3 males and 26 females) had a mean age of 20.30 ± 1.70 years (range = 18–24 years). Both ocular movement and synoptophore data were visibly skewed on a histogram plot, and therefore non-parametric analyses were conducted.

## OCULAR MOVEMENT ASSESSMENT

Twenty-three out of 29 participants (79.31%) had some degree of superior rectus underaction on ocular movement testing in either or both eyes, where 3.45% had a left superior rectus underaction only, 3.45% had a right superior rectus underaction only and 72.41% had a superior rectus underaction in both eyes ranging from –0.5 to –1.5 units. Only 6 participants (20.69%) had no superior rectus underaction in either eye.

It was found that of those with a left superior rectus underaction (alone or alongside a right superior rectus underaction), there was a mean underaction observed in laevo elevation of –0.69 units (range = –1 to –0.5 units) and a median of –1 units (interquartile range (IQR) = –1 to –1 units). Similarly, of the participants with a right superior rectus underaction (alone or alongside a left superior rectus underaction), there was a mean underaction observed in dextro elevation of –0.71 units (range = –1.5 to –0.5 units) and a median of –1 units (IQR = –1 to –1 units). The Wilcoxon test revealed there was no significant difference between left and right superior recti underactions (p = 1.00).

**Figure 1:**
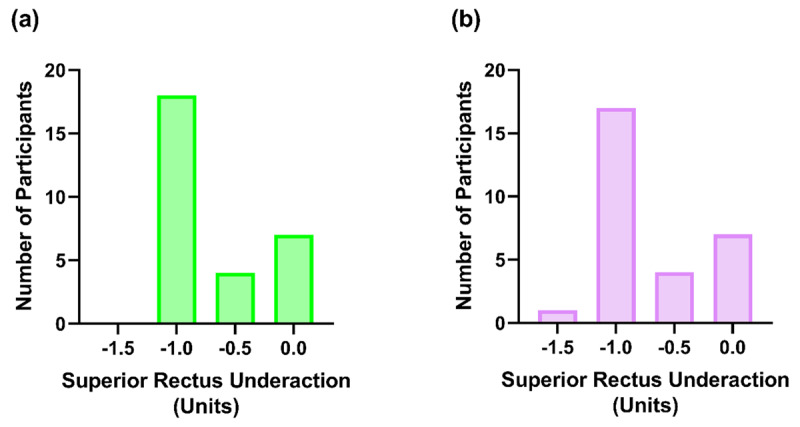
The participant frequency distribution for the left **(a)** and right **(b)** superior rectus underactions found to the nearest +/– 0.5 units during ocular movement testing with the height of the bar representing the number of participants per measurement.

The majority of participants had a superior rectus underaction of –1 units (***Figure 1***). Of these participants, 6.90% had a –1 unit of underaction in the left eye only, 3.45% in the right eye only and 55.17% in both eyes. In addition to this, only one participant demonstrated a superior rectus weakness greater than –1 units in the right eye (in this case –1.5 units), and this was accompanied by a –1 unit of underaction of the superior rectus in the contralateral eye. The expected inferior oblique overactions were observed in both eyes, with a mean overaction of +0.69 units (range = 0 to +1 units) and a median of 1 units (IQR = +0.25 to +1 units).

## SYNOPTOPHORE

The mean horizontal angle in primary position was –0.17 degrees (range = +2 to –2 degrees), with a median of 0 degrees (IQR = –1 to 0.50 degrees). The mean vertical angle fixing left eye in primary position was –0.10 degrees (range = –1 to 0 degrees), with a median of 0 degrees (IQR = –0.25 to 0 degrees). No participants reported torsion.

**Figure 2:**
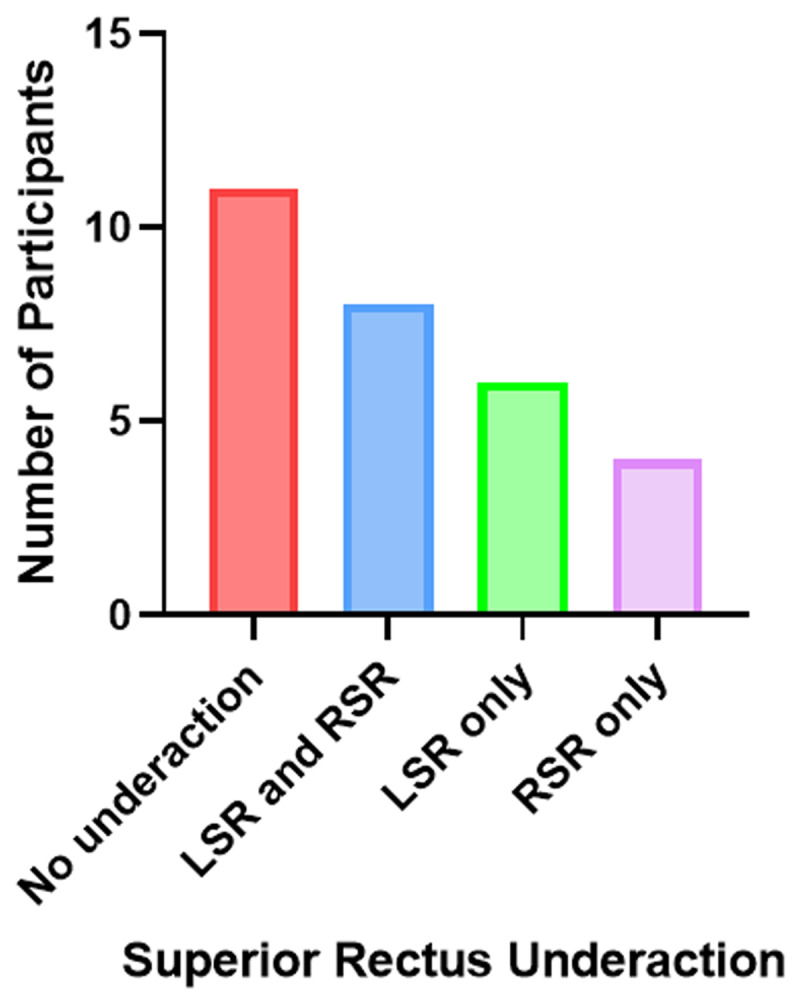
The distribution of superior rectus underactions found across all participants using the synoptophore. LSR = left superior rectus, RSR = right superior rectus.

Eighteen out of 29 participants (62.07%) had some degree of superior rectus underaction where 20.69% had a left superior rectus underaction only, 13.79% had a right superior rectus underaction only and 27.59% had underactions in both eyes, ranging from –1 to –3 degrees. All participants who demonstrated superior rectus underaction on ocular movement testing had a right hyperphoria on the synoptophore in laevo elevation and left hyperphoria in dextro elevation. Only 11 (37.93%) participants were found to have no superior recti underaction in either eye. The relative frequencies of all superior rectus underactions measured to the nearest +/–0.5 degrees can be seen in ***Figure 2***.

Of the participants with a left superior rectus underaction (alone or alongside a right superior rectus underaction), there was a mean underaction measured in laevo elevation of –0.48 degrees (range = –1 to –1 degrees) and a median of 0 degrees (IQR = –1 to –1 degrees). Similarly, those with a right superior rectus underaction (alone or alongside a left superior rectus underaction) had a mean underaction observed in dextro elevation –0.48 degrees (range = –3 to –1 degrees) and median of 0 degrees (IQR = –1 to –1 degrees). The Wilcoxon test revealed no significant difference between left and right superior rectus underaction measurements (p = 1.00).

**Figure 3:**
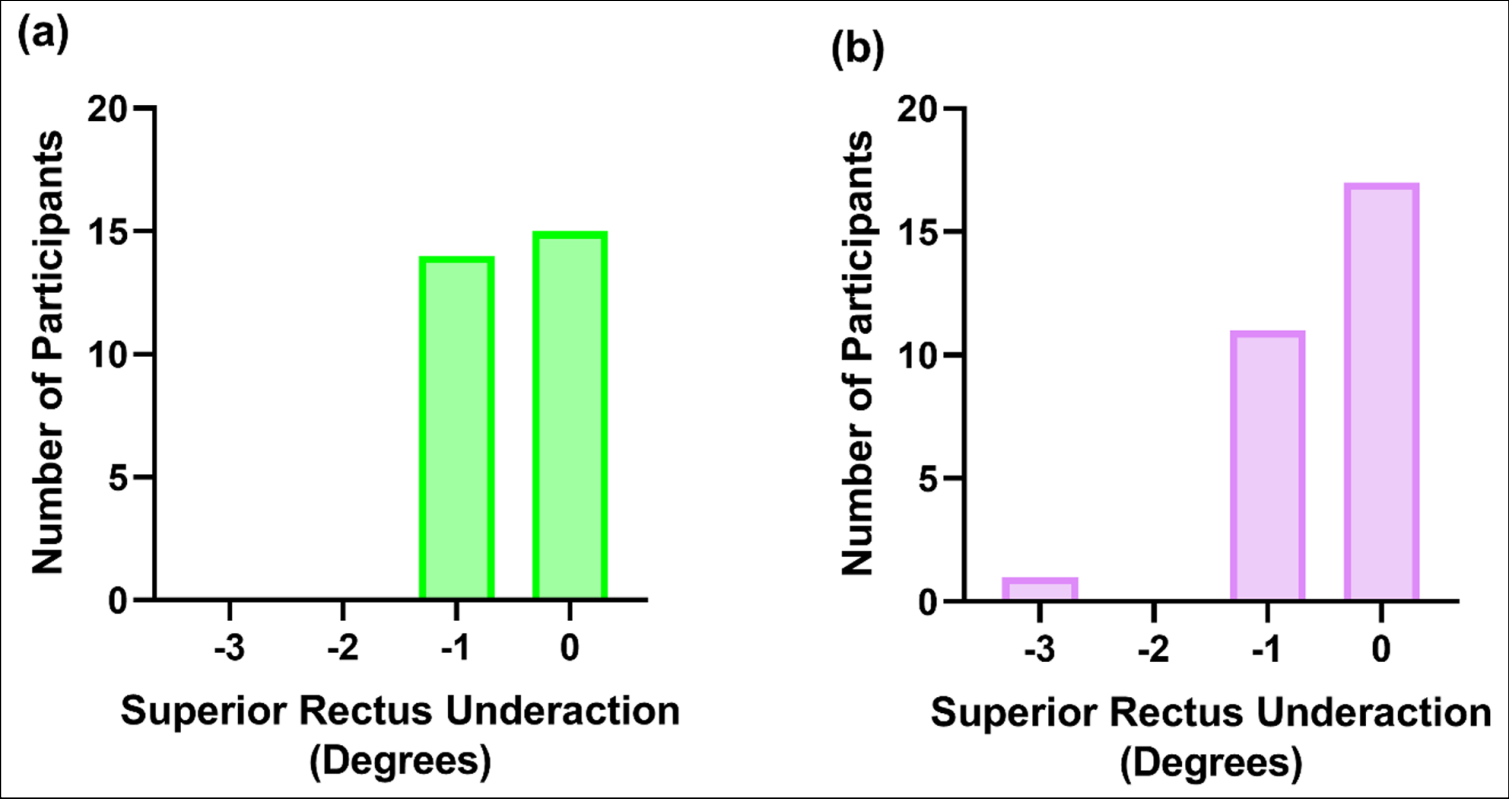
The participant frequency distribution for the left **(a)** and right **(b)** superior rectus underactions found on the synoptophore measured to the nearest +/–0.5 degrees, with the height of the bar representing the number of participants per measurement.

The majority of participants with a superior rectus underaction demonstrated a measurement of –1 degrees (***Figure 3***). Of these participants, 20.69% had this underaction in the left eye only, 13.79% in the right eye only and 24.14% in both eyes. In addition to this, only one participant (3.45%) demonstrated a superior rectus underaction of greater than –1 degrees in the right eye (in this case, –3 degrees) and this was accompanied by a –1 degree underaction of the superior rectus in the contralateral eye.

## COMPARISON OF TESTS

**Figure 4:**
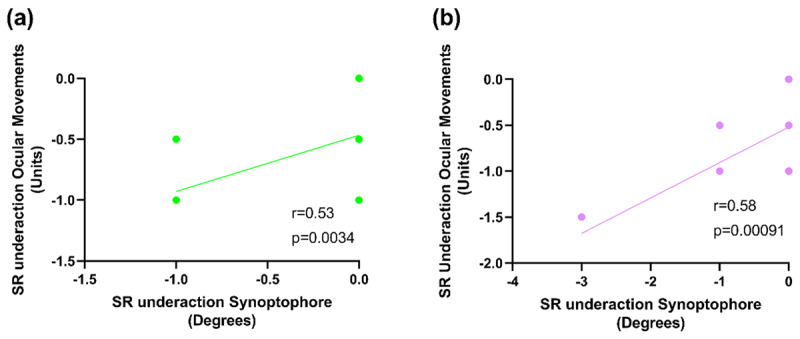
The correlation between the left **(a)** and right **(b)** superior rectus underaction measured using ocular movements and the synoptophore. SR = superior rectus. Note that many of the data points overlap, as illustrated in Figures 1 and 2, and are therefore not visible.

The objective assessment of ocular movements made by the orthoptist is validated by the subjective synoptophore measurements in this study. There was a significant relationship of a moderately positive correlation between the right superior rectus underaction measured using ocular movements and the synoptophore (Rho = 0.58, p = 0.00091, ***Figure 4a***). There was also a significant, moderately positive correlation between the left superior rectus underaction measured using ocular movement and the synoptophore (Rho = 0.53, p = 0.0034, ***Figure 4b***).

## DISCUSSION

The majority of young, healthy students with no known ocular movement defects demonstrated a superior rectus underaction. On ocular movement testing, there was a mean superior rectus underaction of –0.69 and –0.71 units in laevo and dextro elevation, respectively, and a median of –1 units (IQR = –1 to –1 units). On the synoptophore, there was a superior rectus underaction in both eyes of –0.48 degrees and a median of 0 degrees (IQR = –1 to –1 degrees). This supports the theory that it is clinically normal to observe a small degree of superior rectus weakness in healthy young adults.

A greater number of participants were found to have some degree of underaction with ocular movements (23 participants) compared to underactions found with the synoptophore (18 participants). A number of participants were found to have a superior rectus underaction on ocular movements but no underaction on the synoptophore. This may be explained by ocular movement testing being performed in a more extreme position of gaze, than the 20 degrees of elevation at which synoptophore measurements were taken, thus revealing the more subtle underactions. That ocular movement testing revealed the highest frequency of superior rectus underactions is reassuring for clinicians, for whom clinical time is pressurised and would not allow for routine synoptophore testing.

The objective assessment of ocular movements is validated by the subjective synoptophore measurements in this study, with moderate positive correlations in left superior rectus underaction (p = 0.0034) and right superior rectus underaction (p = 0.00091). The correlations found between these tests support the use of ocular movement testing, as routinely conducted by orthoptists. However, Haggerty and colleagues (2005) suggested the ocular movement testing carried out in clinic may be less accurate when quantifying limitations, due to standardisation errors. Their study used the Goldmann Perimeter to measure the monocular upward excursion of the right superior rectus at the exact orientation of the primary field of action (67 degrees from the transverse plane), eliminating the approximate gaze position used when testing ocular movement in free space. One limitation of the current study is that ocular movement assessments were taken at an estimated rather than measured angle and only by one examiner. A future study could both measure the angle and allow multiple assessments to be made by orthoptists with an average then calculated, though observations may slightly differ due to inter-observer variability between orthoptists (Hanif et al. 2009).

The synoptophore was used to measure the subjective underaction of the superior recti over other pieces of equipment, such as the Hess chart, due to the ability to measure slight underactions more accurately, making it more repeatable. The synoptophore scale has 1 degree intervals, whereas each square on the Hess measures 5 degrees, therefore making it difficult to accurately document a small underaction.

Although previous studies have investigated the effect of ageing on the ability to make conjugate upward ocular movements (Clark & Isenberg 2001; Davidson & Knox 2002), to our knowledge, no previous study has collected the normative data for the superior rectus underaction that is believed to be ‘normal’ in young adults on ocular movement testing.

In this study, 62.07% of participants had a superior recti underaction in either eye or both eyes of at least –1 degrees, measured on the synoptophore. On the other hand, Clark and Isenberg (2001) found 89% of healthy patients aged 23 to 84 years had up to 5 degrees of asymmetry between each eye when testing ocular movements in all extreme positions of gaze. The maximum underaction found in this current study was –3 degrees; however, this was only demonstrated in one participant. This may be due to the age of the participants included in each study. Clark and Isenberg included a wider age range; whereas, this study had an age range of 18 to 24 years old, and the significant decline of ocular rotation into elevation with increasing age has been discussed (Chamberlain 1970; Clark & Isenberg 2001; Davidson & Knox 2002). The study by Clarke and Isenberg also used the lateral version light reflex test to measure versions in extreme positions of gaze, where estimations are made based on the decentration of the corneal reflections. Their results were converted into degrees and so cannot be directly compared to results found on the synoptophore due to the inaccuracy when comparing a scaled measurement with an estimated observation.

Chamberlain (1970) found that older participants who had less of a requirement to look in upgaze showed greater limitations of upgaze. Using the Schweiger hand perimeter they found there was 40 degrees upward excursion for 5–14 year olds and 33 degrees upward excursion for 35–44 year olds. Our study tested 18–24 year olds and less degrees into elevation (20 degrees) and found mean underactions of, on average, –0.70 units with ocular movement testing and –0.48 degrees with the synoptophore. Our participants demonstrated a weakness of the superior recti even at a lesser excursion of elevation compared to the findings of Chamberlain. Conversely, results cannot be directly compared as their experiment only examined monocular limitations in direct elevation rather than in laevo and dextro elevation.

The median superior rectus underaction on ocular movements testing was –1 units (IQR = –1 to –1 units) and this would be a good indication of the parameters for what can be considered within normal limits for superior rectus underactions in young, healthy adults. The significant correlation with the synoptophore measurements of left/right hyperphoria on laevo/dextro elevation supports the use of ocular movement testing as routinely conducted by orthoptists. Further work is needed to map the range of superior rectus underactions that can be expected with older age groups as only 18–24 year old students were used within this study. By increasing knowledge of the parameters of what can be considered within normal limits, accuracy of detection of pathology, such as superior rectus palsy, can be achieved. Further work could also explore the underactions in further eccentric positions on the synoptophore and in other, non-orthoptic populations with a greater number of participants.

## CONCLUSION

This study found that most young healthy adults demonstrate small amounts of superior rectus underaction bilaterally on both ocular movement assessment and the synoptophore. On ocular movement testing, –0.70 units of underaction, and on the synoptophore, –0.48 degrees of underaction is the mean level of weakness to be expected. Subtle superior rectus underactions were more frequently revealed by ocular movement than synoptophore assessments. We recommend that superior rectus underactions greater than –1 units for ocular movements and –1 degrees on the synoptophore should be carefully considered for whether further investigation is necessary, together with other important diagnostic information such as asymmetry and LPS weakness.
